# The Roles of CircRNAs in Regulating Muscle Development of Livestock Animals

**DOI:** 10.3389/fcell.2021.619329

**Published:** 2021-03-05

**Authors:** Zhenguo Yang, Tianle He, Qingyun Chen

**Affiliations:** Laboratory for Bio-Feed and Molecular Nutrition, College of Animal Science and Technology, Southwest University, Chongqing, China

**Keywords:** circRNAs, livestock animals, muscle development, co-expression regulatory network, transcription and translation

## Abstract

The muscle growth and development of livestock animals is a complex, multistage process, which is regulated by many factors, especially the genes related to muscle development. In recent years, it has been reported frequently that circular RNAs (circRNAs) are involved widely in cell proliferation, cell differentiation, and body development (including muscle development). However, the research on circRNAs in muscle growth and development of livestock animals is still in its infancy. In this paper, we briefly introduce the discovery, classification, biogenesis, biological function, and degradation of circRNAs and focus on the molecular mechanism and mode of action of circRNAs as competitive endogenous RNAs in the muscle development of livestock and poultry. In addition, we also discuss the regulatory mechanism of circRNAs on muscle development in livestock in terms of transcription, translation, and mRNAs. The purpose of this article is to discuss the multiple regulatory roles of circRNAs in the process of muscle development in livestock, to provide new ideas for the development of a new co-expression regulation network, and to lay a foundation for enriching livestock breeding and improving livestock economic traits.

## Introduction

Circular RNAs (circRNAs) are widely found in eukaryotic cells; they are a special type of nucleotide sequence containing conserved microRNA (miRNA) binding sites (Westholm et al., 2014). Studies show that most of the circRNAs that have been found so far are non-coding RNAs; this kind of circRNA does not have the coding function of linear RNA, but it plays a regulatory role in various life activities, including the process of muscle development in livestock animals (Das et al., 2020). Throughout the research results of the past decade, we find that circRNAs are gradually becoming an indispensable part of the gene regulatory network.

Muscle is an essential component of human and most meat animal bodies, and it plays an important role in providing exercise, maintaining posture, and generating heat (Silva et al., 2019; Janssen et al., 2000). Livestock muscle development is a crucial link to individual growth and development and an important research direction in modern animal science. The condition of muscle development directly exerts an economic effect on animals. Therefore, an in-depth understanding of muscle development is of great significance to the development of animal husbandry.

With the rapid development of molecular genetics and the development of molecular biology technology, *in vitro* cell line culture technology, and gene targeting technology, people have become more and more aware of cell growth and development processes at the molecular level (Li D. et al., 2020). For the past few years, scientists have repeatedly pointed out that circRNAs may be an important biomolecule to understand the mechanism of body development. At the same time, circRNAs play a unique role in regulating human and animal muscle development and its related physiological and pathological processes (Das et al., 2020). Liang et al. identify 149 circRNAs that may be related to muscle growth from 3 skeletal muscles of Guizhou miniature pigs (*S. scrofa*); the gene ontology (GO) and KEGG enrichment analysis of the host gene of the circRNAs indicates that these circRNAs are mainly involved in the growth and development of muscle-related signaling pathways (Liang et al., 2017a). The results show that their host genes are closely related to muscle development, chromatin modification, contraction, ATP hydrolysis-coupled proton transport, and cation homeostasis (Liang et al., 2017a). The above studies show that circRNAs are ubiquitous in muscle and play a critical role in the development process. Therefore, in-depth study of the specific mechanism of circRNAs regulating muscle development has become one of the urgent problems for researchers. Here, we focus on the molecular mechanism and mode of action of circRNA as a competitive endogenous RNA in livestock muscle development. The purpose of this study is to expand researchers’ understanding of the regulation of muscle development by circRNAs, to collect research data for further improvement of the circRNA–miRNA–mRNA (ceRNA) co-expression regulation network, and to provide theoretical support for the improvement of muscle development and economic traits of livestock.

## Overview of Circrnas

### The Discovery of CircRNAs

CircRNA is a kind of closed circular RNA, which can stably exist in the organism, but it does not have the 5′ terminal hat structure and the 3′ terminal poly (A) tail structure (Westholm et al., 2014). As early as the 1970s and 1980s, researchers proposed in Nature and PANs that circRNA is a kind of covalently closed circular RNA molecule discovered in plant viroids and eukaryotic cells (Sanger et al., 1976; Hsu and Coca-Prados, 1979; Kos et al., 1986). Later, Danan et al. (2012) found that some non-coding RNAs, snoRNAs, and RNase P RNAs could form circRNAs in archaea. Although these early studies clearly document the existence of circRNA molecules, their potential impact was underappreciated. With the advent of some advanced RNA sequencing techniques and methods for calculating non-polyadenylate RNA transcription, more and more circRNAs have been found in paleontological, nematode, zebrafish, mouse, and human cells (Zhang et al., 2014), and regulation of circRNA levels can lead to a variety of molecular and physiological phenotypic changes, which include effects on growth and development (Liu B. et al., 2019), the nervous system (Irie et al., 2019), innate immunity, microRNAs (Yang et al., 2017), and many disease-related pathways (Du et al., 2018). As a result, circRNA became a hot spot.

### The Classification of CircRNAs

In order to carry out follow-up research in a more organized way, scholars depend on the genomic loci and the relationship with the connected parental transcript; circRNAs are categorized into five types: exonic (Danan et al., 2012; Salzman et al., 2012; Memczak et al., 2013; Li X. et al., 2019), intronic (Zhang et al., 2013; Das et al., 2019a,b), sense overlapping (Humphreys et al., 2019; Patop et al., 2019), antisense, and intergenic (Qu et al., 2015; Wang et al., 2016). Cao et al. (2018) found 886 circRNAs in the form of introns and exons in the sheep skeletal muscle circRNA library—most of which interact with muscle-specific miRNA involved in muscle growth and development, especially circ776. It is worth noting that there are few reports on other types of circRNAs regulating muscle development. Therefore, this is also a place where we need further study and breakthroughs.

### The Biogenesis of CircRNAs

**FIGURE 1:**
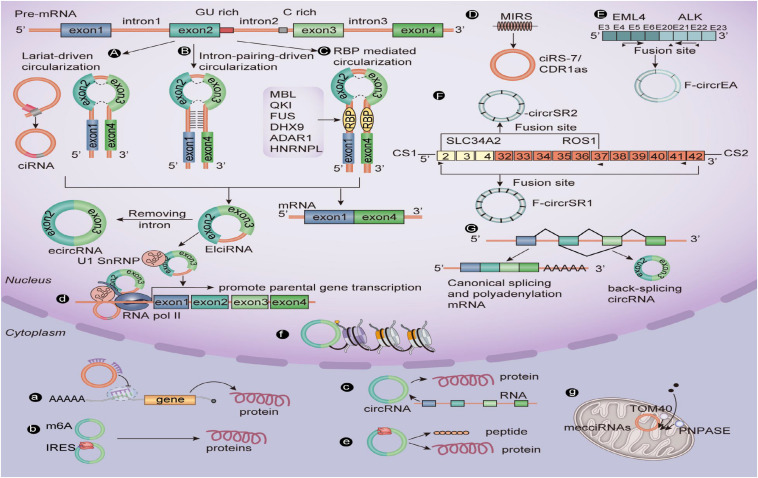
The biogenesis mechanisms and biological roles of circular RNAs. **(A)** Lariat-driven circularization. **(B)** Intron pairing–driven circularization. **(C)** RBP-mediated circularization. **(D)** Repetitive MIR-mediated circularization. **(E)** EML4-ALK fusion gene–mediated circularization. **(F)** SLC34A2-ROS1 fusion gene–mediated circularization. **(G)** Interfering with gene-mediated circularization that regulates transcription. (a) CircRNAs can sponge miRNAs. (b) A few circRNAs containing the m^6^A motif or IRES can encode functional proteins. (c) CircRNAs splice with linear RNA to promote the expression of mRNA. (d) EIciRNA can interact with U1 small nuclear ribonucleoproteins and then increase the transcription of their host genes by binding with RNA pol II; ciRNA, and the RNA pol II complex can directly interact and play a role in regulating parental gene transcription. (e) CircRNAs can be translated into peptides or proteins. (f) CircRNA in some nuclei is involved in histone modification. (g) Mitochondrial-derived circRNA can regulate the entry of proteins into mitochondria under the interaction of TOM40 and PNPASE. CiRNA, Circular intronic RNA; EcircRNA, Exonic circRNA; EIciRNA, Exon–intron circRNA; RBP, RNA-binding protein; RNA pol II, RNA polymerase II.

The difference in structure between circRNA and linear RNA means that they are formed in different ways and have different biological functions (Figure 1). Li X. et al. (2019) prove for the first time that the assembly mechanism of the “intro-definition” and “exon-definition” E complex can exist from the point of view of structural biology. On this basis, they point out that some E complexes assembled on the middle exons of yeast EFM5 or HMRA1 can be chased into circRNA, but this requires exons long enough to achieve this, and most eukaryotic circRNAs are catalyzed by classical splice bodies or group I/II ribozymes (Lasda and Parker, 2014; Chen and Yang, 2015). With reference to the existing research, we summarize and sort out the formation of circRNAs: First, the lasso structure drives cyclization, and the lasso structure is a by-product of exon hopping. After the intron in the lasso structure is removed, exons can be connected to form circRNAs. The second is intron pairing driving cyclization, and there is an intron with a reverse complementary sequence at both ends of the ring-shaped exon. The pairing mediation of the reverse complementary sequence of the intron can make the splicing donor and splice recipient of the exon spliced into a ring closer to each other in space, thus forming circRNAs (Li X. et al., 2019). Besides this, the RNA binding protein (RBP) is an important factor in regulating the production of circRNA. RBP can specifically bind to the flanking introns at both ends of RNA, acting as an RBP while narrowing the distance between the splice recipient and the donor, resulting in the formation of circRNAs (Naqvi et al., 2019; Pagliarini et al., 2020; Zhao et al., 2020). It is well-known that CDR1as, as an antisense transcript of cerebellar degeneration associated protein 1, can be used as a specific circRNA of miR-7, so it is also known as CIRS-7. However, it is surprising that ciRS-7/CDR1as biosynthesis in circRNAs is mediated by mammalian scattered repetitive elements mammalian-wide interspersed repeats (MIRs) (Yoshimoto et al., 2020). In addition, a recent study shows that m^6^A modification can promote the production of circRNAs carrying an open reading frame (ORF) during the development of male germ cells in mice (Tang et al., 2020). Tan et al. (2018) report that the EML4-ALK fusion gene can form F-circrEA. Later, Wu et al. (2019) found that SLC34A2-ROS1 can form F-circrSR1 and FmurcircrSR2 by the fusion gene, and both of them may be used as diagnostic markers of lung cancer. Also, some scholars find that genes that interfere with transcriptional termination contribute to the production of transcriptional read-through products and promote the production of downstream gene-derived circRNAs (Liang et al., 2017; Chen S. et al., 2019). From this point of view, the formation of circRNAs is affected by many biological factors. Therefore, the formation of circRNAs still needs in-depth study by researchers.

### The Biological Characteristics of CircRNAs

Many studies show that circRNA is a structure with a missing 5′ end cap and 3′ end poly (A) tail, so it is not easy for circRNAs to be degraded by exonuclease RNAseR (Suzuki et al., 2006; Suzuki and Tsukahara, 2014; Li X. et al., 2019). Further, due to circRNAs being highly conserved in many species, such as humans, nematodes, zebrafish, and mice (Salzman et al., 2012; Li X. et al., 2019), only a small number of them can evolve and change rapidly (Zhang et al., 2013, 2014). Some studies point out that the number of circRNAs found in eukaryotic cells has been as high as more than 20,000 (Cocquerelle et al., 1993; Westholm et al., 2014); a few are formed by direct cyclization of introns, and most of them come from exons (Liu et al., 2020a). In addition, as a kind of non-coding RNA, circRNA can only regulate the formation of proteins at the transcriptional or post-transcriptional level (Westholm et al., 2014), and only a few of them can regulate life activities by encoding proteins (Pamudurti et al., 2017; Yang et al., 2018). Medical researchers believe that circRNAs have certain tissue and disease specificity and have guiding significance for the treatment of many diseases (Cocquerelle et al., 1993).

### The Biological Function of CircRNAs

In the past decade, research results have shown that circRNAs can directly or indirectly participate in the process of gene expression, such as RNA translation, miRNA bait, RNA translation, protein–protein interaction, and so on (Westholm et al., 2014; Pamudurti et al., 2017; Yang et al., 2018) (Figure 1). Based on the results of previous studies, we summarize the biological functions of circRNAs: (1) CircRNAs can be used as an miRNA sponge to regulate the stability of related mRNAs or protein formation. For example, circHIPK3 acts as a sponge of miR-7 in CRC when the co-expression of miR-7 and circHIPK3 promotes the proliferation of colorectal cancer cells (Zeng et al., 2018). (2) A few circRNAs can encode functional proteins when they contain the m^6^A motif or IRES. For example, studies show that some circRNAs can carry longer ORFs, and the initiation codon of these ORFs is modified by m^6^A and binds to ribosomes to form functional proteins (Zhao et al., 2018; Tang et al., 2020). (3) CircRNAs can be spliced with linear RNA and promote the expression of linear mRNA. For example, complementary pairing of CDR1as and CDR1mRNA can enhance the stability of CDR1mRNA (Rong et al., 2017). (4) EIcircRNA or ciRNA interacts with Pol II and U1 snRNP at the promoter of the parent genes, thus promoting the transcription of the parent genes. For example, circEIF3J and circPAIP2 regulate gene expression by forming complexes with U1 snRNP and Pol II, which bind to the promoter region of the host gene (Li D. et al., 2020). (5) Some circRNAs can be used as translation templates for proteins and peptides. For example, some ribo-circRNAs use the start codon of the host mRNA and bind to membrane-related ribosomes to participate in circRNA translation (Granados-Riveron and Aquino-Jarquin, 2016; Pamudurti et al., 2017). (6) CircRNAs in some nuclei are also involved in histone modification (Burd et al., 2010; Kotake et al., 2011) and RNA maturation (Holdt et al., 2016). CircRNAs (mecciRNAs) from humans, and mouse mitochondria can enter mitochondria by interacting with TOM40 and PNPASE (Liu et al., 2020b).

### The Degradation of CircRNAs

CircRNAs maintain their cellular homeostasis by highly dynamic and tightly regulated biogenesis and degradation, thereby exerting proper biological functions. Compared with the biogenetic mechanism of circRNA biogenesis, the specific pathway by which cells eventually degrade circRNAs is still in need of continued study. Some studies show that miRNAs may initiate the degradation of circRNAs through Ago2-mediated cleavage. For instance, as the target of CDR1as/ciRS-7, miR-671 can perfectly load Ago2 into CDR1as/ciRS-7, which leads to the cleavage of Ago2 in the nucleus and the subsequent dissolution of RNA in the outer nucleus. However, it is not known whether other miRNAs can regulate the degradation of circRNAs by perfectly matching them with Ago2 (Hansen et al., 2011). A recent study has shown that m^6^A can mediate circRNA degradation. Park et al. (2019) point out that, when m^6^A carrying circRNA is used as a marker, m^6^A read–write protein YTHDF2 and linker protein HRSP12 can be recruited. As a bridge between YTHDF2 and endoribonuclease RNase P/MRP, HRSP12 binds directly to the GGUUC motif on circRNAs, which leads to the initiation of RNase P/MRP and the gradual degradation of circRNAs. In addition, Fischer et al. (2020) find a degradation mechanism of RNA (including circRNAs). He also points out the specific mechanism of selective degradation of high-structure RNA under normal conditions. High overall structure circRNA decay is regulated globally by two RNA binding proteins, UPF1 and G3BP1 (Chen et al., 2020). Because this pathway perceives the whole RNA structure rather than a linear first-order sequence, it is called structure-mediated RNA decay (SRD). Because mammalian RNA decay pathways are widely linked to translation (Tuck et al., 2020), it is also worth exploring whether circRNAs targeted by SRD have potential encoding peptides. Furthermore, Liu C. X. et al. (2019) discovered an endonuclease RNaseL that can degrade circRNAs in a full range, which seems to increase researchers’ understanding of the mechanism of circRNA degradation.

## General Situation of Muscle Development of Livestock Animals

Muscle development is a very complex biological process, which mainly depends on the proliferation and hypertrophy of muscle fiber cells (Molkentin and Olson, 1996). Studies show that the number of muscle fibers increases only before birth and does not change much after birth, and the growth of muscle fibers depends on the hypertrophy of muscle fibers (Christ and Brand, 2004). The hypertrophy of muscle fibers includes two aspects: One is the increase of myofibrils, and the other is the increase in the number of nuclei in muscle fibers (Berger et al., 2015). Muscle development is a multistep process regulated by multiple genes, and it is not only regulated by a variety of myogenic regulatory factors, including myogenin (MyoG), myogenic regulatory factor-4 (MRF-4), myosin heavy chain (MyhC), myoblast regulatory factor family myogenic factor-5 (Myf-5), myostatin (MSTN), myocyte enhancer factor2A-D (MEF2A-2D), myogenic differentiation antigen (MyoD), and paired box (Pax) family members Pax 3 and Pax 7 (Chen et al., 2020), but it is also regulated by other related genes, such as insulin-like growth factor-I (IGF-I), insulin-like growth factor-II (IGF-II), forkhead box transcription factor O1 (FoxO1), and mammalian target of rapamycin (mTOR) (Wan et al., 2016). Furthermore, studies confirm that the genes that affect muscle development have two regulatory effects: One is positive promotion; the other is reverse inhibition (McPherron and Lee, 2002; Chen P. R., 2019; Chen et al., 2020). These growth factors play a unique role in regulating muscle development and can regulate cell proliferation, apoptosis, sarcomere activation, and muscle-specific genes at many sites in the muscle (Doynova et al., 2017; Hernández-Hernández et al., 2017) pedigree.

**FIGURE 2:**
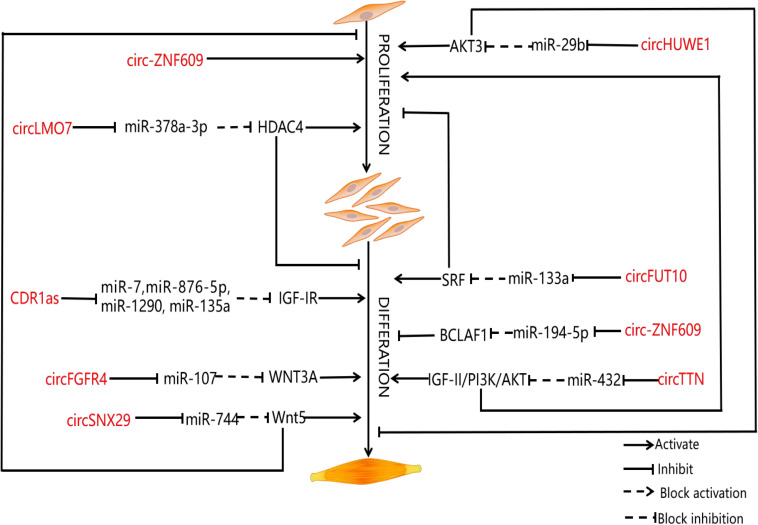
CircRNAs involved in muscle development and their function. Schematic representation of the role of circRNAs in muscle proliferation and differentiation. The diagram shows various circRNAs that regulate the proliferation and differentiation of myoblasts. The circRNAs are represented in red.

Exploring the regulatory mechanism of muscle cell proliferation and differentiation is one of the research hot spots in developmental biology in recent years. As an important economic trait, the muscle development of livestock has been paid more and more attention by researchers. CircRNAs have been widely studied in the related fields of human medicine and bioinformatics, which provides a new idea for exploring the construction of regulatory networks of circRNAs in animal husbandry. As was mentioned earlier, muscle development is a complex biological process that is affected by many factors. Although researchers have conducted extensive and in-depth studies on circRNAs, some researchers noticed that circRNAs can further guide the process of muscle development by binding to miRNAs or regulating the expression of genes related to muscle development at the transcriptional level (Zhang et al., 2019). Based on previous studies, we summarize the unique role of circRNAs in muscle development (as shown in the Table 1 and Figure 2).

**TABLE 1:** CircRNAs involved in muscle development and their function.

**circRNAs**	**Parental gene**	**Organism**	**Biological roles**	**Target miRNA(s)**	**miRNAs target**	**References**
circ-ZNF609	ZNF609	Homo sapiens	Inhibits differentiation, promotes proliferation; can be translated	miR-194-5p	BCLAF1	Legnini et al., 2017
circLMO7	LMO7	Bovine	Inhibits differentiation, promotes proliferation of primary bovine myoblasts and protects them from apoptosis	miR-378a-3p	HDAC4	Wei et al., 2016
circFUT10	FUT10	Cattle	Reduces proliferation and facilitates differentiation of bovine myoblasts	miR-133a	SRF	Li et al., 2018
circSNX29	SNX29	Bovine	Promote myoblast differentiation and inhibit cell proliferation	miR-744	Wnt5a CaMKIIδ and Ca^2+^	Peng et al., 2019
circFGFR4	FGFR4	Bovine	Promote cell differentiation	miR-107	WNT3A	Li et al., 2018
circHUWE1	HECT, UBA and WWE	Bovine	Promotes myoblast proliferation and inhibits differentiation	miR-29b	AKT3	Yue et al., 2020
bta_circ_03789_1, bta_circ_05453_1	ND	Cattle	May regulate the IGF-IR gene by regulating the miRNAs associated with the longissimus dorsi muscle, and finally regulate muscle development	miR-133b and miR-664a	IGF-IR	Yan et al., 2020
circTTN	TTN	Bovine	Promoted proliferation and differentiation	miR-432	IGF-II, PI3K, AKT	Wang et al., 2019
circ776	ND	Sheep	Involved in muscle cell development and signaling pathway	miR-208	ND	Cao et al., 2018
circ_0001573, circ_0001554, circ_0013564	ND	Pig	Regulation of muscle fiber transformation through mi-499-5p	miR-499-5p	KCNQ1, MRAS and SERTM1	Li B. et al., 2020
CDR1as	Human X Chromosome	Goat	Promotes myoblast differentiation	miR-7, miR-1290, miR-876-5p, miR-135a	IGF-1R	Sun et al., 2017

*ND, not determined.*

It is reported that the number of circRNAs in animal muscles and muscle cells ranges from 2,000 to 37,000 (Abdelmohsen et al., 2015; Li et al., 2017; Liang et al., 2017b; Wei et al., 2017; Ouyang et al., 2018; Zhang et al., 2018). Li et al. (2017) first used RNA sequencing to detect 6,113 circRNAs from the longissimus dorsi muscle of sheep. The researchers found 12,000 circRNA expressions during muscle aging in monkeys by RNA sequencing (Abdelmohsen et al., 2015). The study found that circRNAs regulated the growth and development of porcine skeletal muscle and the transformation of muscle fiber types at the age of 0–30 days; at the age of 30–240 days, circRNAs regulated the glucose metabolism and calcium signal of porcine skeletal muscle (Liang et al., 2017b). Many reports point out that circRNAs play a unique and irreplaceable role in guiding animal muscle development (Abdelmohsen et al., 2015; Li et al., 2017; Liang et al., 2017b; Wei et al., 2017; Ouyang et al., 2018; Zhang et al., 2018). Liang et al. (2017b) constructed the first miniature pig circRNA database and point out that ssc-ciR-02753, ssc-ciR-03065, ssc-ciR-03066, ssc-ciR-03069, ssc-ciR-04335, ssc-ciR-04348, ssc-ciR-04349, ssc-ciR-04353, and ssc-ciR-04359, can regulate porcine muscle development by affecting cell proliferation and fusion. It is reported that many circRNAs contain binding sites, such as miR-1, miR-133, miR-206, miR-29, miR-378, miR-431 (Ebbesen et al., 2017), miR-7 (Li L. et al., 2019), miR-135a (Greco et al., 2009), miR-1290 (Ng et al., 2015), and miR-876-5p (Cook et al., 2015). Interestingly, these miRNAs are involved in cell proliferation, differentiation, and signal transduction during myogenesis. It lays a theoretical foundation for the construction of a co-expression network of circRNAs regulating the muscle development of livestock. Combined with human medical research, it is not difficult to see that circRNAs and miRNAs can bind and regulate the expression of downstream mRNAs (that is, the ceRNA co-expression regulatory network) in a certain way. A lot of evidence also shows that the muscle development of livestock is indeed regulated by both circRNAs and miRNAs (Abdelmohsen et al., 2015; Li et al., 2017; Liang et al., 2017b; Wei et al., 2017; Ouyang et al., 2018; Zhang et al., 2018). In addition, a very small number of circRNAs can regulate muscle development in livestock at the transcriptional and translational levels (Shen et al., 2019). However, there is almost no relevant research in this field at present. Therefore, it is necessary for us to pay close attention to the related regulatory mechanisms of circRNAs on muscle development in different ways.

## Circrnas Regulate Muscle Development of Livestock Animals

### CeRNA Co-expression Network Regulates Muscle Development of Livestock Animals

Muscle development of livestock is an important economic character in the development of animal husbandry. Despite the controversy surrounding the ceRNA hypothesis, large amounts of experimental results show that circRNAs can regulate the expression of mRNAs related to the muscle development of livestock through specific miRNAs (Qian et al., 2017). Therefore, researchers have evaluated multiple co-regulatory relationships during muscle development. Among them, Yue et al. (2020) verify the interaction among circHUWE1, miR-29b, and AKT3 with the help of bioinformatics, double luciferase report analysis, and AGO2-RNA immunoprecipitation (RIP) and point out that circHUWE1 can directly interfere with the ability of miR-29b to release AKT3 inhibition and finally activate the AKT signal pathway, thus promoting the proliferation of bovine myoblasts and inhibiting apoptosis and differentiation of bovine myoblasts. For this reason, Yue et al. (2020) build upon previous research results to construct a ceRNA co-expression regulatory network, which not only provides a good idea for the study of muscle development, but also expands our understanding of the function of circRNAs. KEGG pathway analysis shows that hosting genes of circRNAs are related to the muscle development pathway, including the mammalian target of rapamycin signaling pathway, Wnt signaling pathway, MAPK, and transforming growth factor-β signaling pathway (Li et al., 2017). Yan et al. (2020) identify a total of 5,177 circRNAs in the longissimus dorsi samples of Kazakh cattle and Xinjiang brown cattle by establishing a RNA sequence library; of these, 46 are differentially expressed. The identification of differentially expressed genes shows that the process of muscle development is related to differentially expressed circRNAs. In addition, miRanda predictions show that there are 66 interactions between 65 circRNAs and 14 miRNAs. For this reason, Yan et al. (2020) also establish a co-expression network. Further, some miRNAs known to be involved in myoblast regulation, such as miR-133b and miR-664a, are identified. Sun et al. (2017) study the differentially expressed coding genes, lncRNAs, circRNAs, and miRNAs, in muscle tissue of Lantang and Landrace pigs. The results show that there are 1,401 high expressions of circRNAs and 2,959 low expressions of circRNAs in Lantang pig, of which 236 circRNAs are closely related to muscle development, and 40 circRNAs regulate muscle development by participating in a miRNA-mediated ceRNA regulatory network. There are 6,113 differentially expressed circRNAs in the longissimus dorsi of Kazakh sheep at both embryonic and adult stages, and the maternal genes of circRNAs are mainly enriched in the signal pathways related to muscle growth and development. Among them, oar_circ 0000385, oar_circ_0000582, and oar_circ_0001099 have multiple binding sites on miRNAs (miR-143, miR-133, and miR-23) related to muscle development (Li et al., 2017). Wei et al. (2017) detect the circRNA expression profile of bovine skeletal muscle at two developmental periods (embryonic and adult longissimus) for the first time and point out that the overexpression of circLMO7 can competitively absorb miR-378a-3p when the expression of miR-378a-3p is downregulated; the target gene hdac4 is activated, thus promoting muscle cell proliferation and inhibiting muscle cell differentiation. After that, Li et al. (2018) find that circFGFR4 and miR-107 are highly expressed in Qinchuan cattle at the embryonic stage (90 days) and adulthood (24 months old) longissimus dorsi, and circFGFR4 could adsorb miR-107. MiR-107 can weaken the expression of Wnt3a by binding to overexpressed circFGFR4. It can be seen that Wnt3a, as the target of miR-107, plays an important role in inhibiting myotube formation and protecting myoblast apoptosis. To sum up, circFGFR4 can be used as an miR-107 sponge to eliminate the inhibitory effect of miR-107 on the expression of Wnt3a and the differentiation of bovine primary myoblasts. The differential expression of circTitin (circTTN) in bovine skeletal muscle between fetal and adult bovine muscle tissue and the overexpression and inhibition of circTTN induced its promoting effect on the proliferation and differentiation of bovine primary myoblasts because miR-432, the target gene of circTTN, is the regulator of IGF-II. Wang et al. (2019) point out that circTTN can activate the IGF-II/phosphatidylinositol 3-kinase (PI3K)/AKT signal pathway through competitive binding with miR-432, which promotes the proliferation and differentiation of bovine myoblasts. Li et al. (2018) find that circFUT10 in adult bovine muscle can reduce its inhibition on target genes by competitive binding to miR-133a, and the expression of MyHC, MyoD, and MyoG related to muscle development changed synchronously with that of circFUT10 at the mRNA and protein levels. Overexpression of circ-FUT10 can promote MyHC expression, induce myoblast apoptosis, and promote myoblast differentiation. On the contrary, circZfp609 binding to miR-194-5p inhibits the expression of bcl2-related transcription factor 1 (BCLAF1), which also affects the expression of Myf5 and MyoG, and inhibits myoblast differentiation (Li et al., 2018). CircSNX29 exists widely in bovine primary myoblasts, but its expression level in embryonic skeletal muscle is significantly higher than that in adult skeletal muscle. Peng et al. (2019) find that overexpression of circSNX29 promotes myoblast differentiation and inhibits cell proliferation while interfering with circSNX29 inhibits myoblast differentiation and promotes proliferation. Subsequently, using RNAhybrid for bioinformatics prediction, it was found that circSNX29 may adsorb to miR-744 with 9 potential binding sites. Using a double luciferase report assay, the results show that circSNX29 could directly bind to miR-744 competitively and effectively reverse the inhibitory effect of miR-744 on Wnt5a and CaMKIIδ. Importantly, through KEGG pathway enrichment analysis, Western blotting, a calcium fluorescence probe, and CamKII activity detection, it is found that overexpression of Wnt5a and circSNX29 activate the non-classical Wnt/Ca^2+^ pathway by increasing the activity of CamKII kinase and the phosphorylation level of PKC and then regulate the proliferation and differentiation of bovine myoblasts. These results are helpful to further understand the role of circRNAs and miRNAs in myogenesis. The study further shows that muscle development is more efficient in embryo than in adulthood. Interestingly, Von et al. point out that Wnt5a and CaMKIId are the targets of miR-744, and the expression of miR-744 leads to the activation of the atypical Wnt pathway by inhibiting the expression of Wnt5a and CaMKIId (von Maltzahn et al., 2012). From this, we can see that circSNX29 acts as an miR-744 sponge to upregulate the expression of CaMKIId and Wnt5a through activating the Wnt pathway and promotes myoblast differentiation. In addition, it is reported that circRNA9210-miR-23a-MEF2C and circRNA290-miR27b-Foxj3 networks play a unique role in regulating the conversion of muscle fiber types in porcine skeletal muscle (Shen et al., 2019). Li et al. (2017) find a total of 5,086 differentially expressed circRNAs in the RNA sequences of sheep adult longissimus dorsi (LDM-A) and longissimus dorsi (LDM-E) of which 2,146 are downregulated and 2,940 are upregulated. The results of real-time quantitative PCR show that the expression of circRNA 0000552, circRNA 00002456, circRNA 00004666, circRNA00004676, and circRNA 00004690 in LDM-E is relatively higher than that of LDM-A. The expression of circRNA 0003451, circRNA 0005243, circRNA 0005250, and circRNA 0005256 in LDM-A is relatively higher than that in LDM-E. Thus, the differential expression of circRNAs in sheep muscle is proved. Cao et al. (2018) extracted 75.5 million sequences from the sheep skeletal muscle RNA gene bank. These sequences were mapped to 729 genes in the reference genome of sheep, containing a total of 886 circRNAs. Reverse transcription PCR and DNA sequencing analysis confirm the existence of many kinds of circRNAs and the resistance of sheep circRNAs to RNase R digestion. Finally, Cao et al. (2018) first used RNA-seq to study circRNAs in the longus dorsi muscle of sheep before and after delivery. A total of 6113 circRNAs were detected, of which some circRNAs (circRNA100, circRNA108, circRNA205, circRNA606, circRNA678, circRNA744, and circRNA776) contained at least two conservative targets of miRNAs related to muscle development (miR-29b, miR-133, miR-208, and miR-499, respectively). Thus, it can be seen that most circRNAs interact with muscle-specific miRNAs and then jointly regulate the process of muscle development. The results of GO and KEGG enrichment analysis show that the host gene of circRNAs is involved in muscle cell development and signal transduction (Cao et al., 2018). For this reason, Cao et al. (2018) establish a relatively complete ceRNA network that contains a large number of potential functional circRNA (circRNA 0000385, circRNA 0001099, and circRNA 0000582) and its predicted miRNA targets and downstream regulatory genes. At present, these regulatory networks are an important source of ideas for us to study muscle development. This information may help us to further explore the unique role of circRNAs in muscle development.

As we all know, circRNA CDR1as (CDR1as or CiRS-7) is an antisense transcript of cerebellar degeneration associated protein 1, but in fact, CDR1as is also considered to be related to miRNAs related to muscle development (Geng et al., 2016; Sang et al., 2018). Although CDR1as was identified as the specific circular RNA of miR-7, other miRNAs, such as miR-135a, miR-876-5p, and miR-1290, are also shown to be CDR1 response elements (Geng et al., 2016; Sang et al., 2018). It is worth noting that CDR1as-responsive miRNA and its targeted muscle-derived genes, such as IGF-IR, N-cadherin, ABCG2, WNT5A, EGFR, FAK, and CCNE1, play a key role in normal conditions and muscle diseases, such as DM1, FSHD, and IIM, which makes CDR1 potentially an important regulatory factor in muscle (Geng et al., 2016; Sang et al., 2018; Kyei et al., 2020). First of all, Li L. et al. (2019) discovered that MyoD promotes CDR1as by binding on the CDR1as 5′ flank region; however, the overexpression or knockout of CDR1as can significantly induce or hinder the process of muscle differentiation. Second, CDR1as can reduce the downregulation of IGF-IR induced by miR-7 through competitive binding to miR-7, thus activating muscle differentiation in goat metaphase. The above results further indicate that CDR1as plays an irreplaceable role in the regulation of muscle development. At the same time, these potential CDR1as/miRNAs/mRNA regulatory networks provide a basis for further study of the function of CDR1as in muscle development and other life activities and processes.

In general, these results show that circRNA is a key factor that cannot be ignored in the process of muscle development; it can compete for endogenous miRNAs to form a circRNA–miRNA complex and further relieve the inhibitory effect of miRNAs on mRNA. However, the interaction between circRNAs and endogenous miRNAs needs to be further verified because, in some special cases, the number of miRNA sites that bind to a specific circRNA is limited, and these sites are specific in different species or tissues, so the ceRNA regulatory network is not the whole content of muscle development.

### CircRNA Regulates Muscle Development in Livestock Animals at the Transcriptional Level

Because there is no biological original to guide coding protein in the special structure of circRNA, circRNA is considered to be a non-coding RNA in most cases. However, recently, researchers are increasingly finding that a very small number of circRNAs have the ability to encode proteins (Li and Lytton, 1999; Legnini et al., 2017; Yang et al., 2018; Liang et al., 2019). Shen et al. (2019) point out that circRNA41, circRNA69, and circRNA153 differentially expressed in porcine skeletal muscle during oxidation and glycolysis are transcribed from MyH1 (encoding MyHC-2X protein), MyH7 (encoding MyHC-β protein), and MyH2 (encoding MyHC-2A protein) genes, respectively. These results indicate that circRNAs play a unique role in regulating the heterogeneity of muscle fiber types, but at present, the research in this area has not been reported. In addition, previous studies point out that the protein encoded by circRNAs depends on the ORF on the sequence. However, the circRNAs that actually have the function of coding protein should have many necessary conditions at the same time, such as the internal ribosome entry sites (IRES), the biological element activated by translation, and the biological element for detecting protein products (Wang and Wang, 2015; Legnini et al., 2017; Pamudurti et al., 2017). A typical example is circRNA ZNF609, which is one of the earliest endogenous circRNAs that can be translated into a protein driven by RES and regulating myogenesis. Circ-ZNF609 itself does not possess the factors required for cap structure and polyadenosine transcription translation, but it can be involved in muscle cell development by initiating translation under cis-acting elements through a mechanism independent of the cap structure in response to different cellular stresses (Legnini et al., 2017). In addition, a recent study finds that circSamd4 is related to Pura and PURB during muscle development; as myogenic inhibitors, Pura and PURB can inhibit the transcription of the myosin heavy chain (MHC) protein family. Silencing CircSamd4 enhances the binding of PUR protein to the MHC promoter, and overexpression of circSamd4 interfers with the binding of pur protein to the MHC promoter, indicating that circSamd4 could bind to PUR protein and prevent it from interacting with DNA. When using mutant circSamd4 without a PUR binding site, these effects were canceled. In other words, the binding of PUR protein to circSamd4 can promote muscle development by reducing MHC transcription (Pandey et al., 2020). Song et al. (2020) identify 197 differentially expressed circRNAs in the gastrocnemius of Duchenne muscular dystrophy (DMD) mice and predicted their protein coding ability according to the Nmurine 6-methyladenosine motif and ORF of circRNAs. Among them, 189 circRNAs were predicted to have protein coding potential, and 98 circRNAs may be translated into peptides containing 150 or more amino acids, indicating that circRNAs may play a key role in the pathophysiological mechanism of DMD. CircFAM188B contains an ORF that can be translated as circFAM188B-103aa during the skeletal muscle development of broilers to promote the proliferation of chicken SMSC (Yin et al., 2020). To sum up, although researchers have gradually deepened their understanding of circRNAs, there are still few studies on circRNAs regulating muscle development by encoding proteins.

### CircRNAs Directly Regulate Muscle Development Through mRNA

As we mentioned earlier, muscle development in livestock is a complex physiological process, and different kinds of circRNAs play different roles in muscle development. Ling et al. (2020) find that cluster16 circRNAs are highly expressed in the early and late stages of muscle development of Anhui white goat (AWG) embryo and are directly involved in the Wnt signal pathway, AMPK signal pathway, and so on. It can be seen that circRNAs can directly regulate the muscle development of livestock. In addition, circQKI (as well as QKI mRNA) depletion is demonstrated to have a negative effect on myoblast differentiation, indicating that both the circRNA and its linear counterpart cooperate in this process. By contrast, although BNC2 mRNA depletion causes an increase in myotube formation, knockdown of its circular counterpart has no effect on differentiation. Interestingly, circBNC2 expression during myoblast differentiation increases at the expense of the corresponding mRNA, suggesting that circBNC2 could contrast the expression of the anti-differentiative BNC2 mRNA (Legnini et al., 2017). Similarly, circEch1 is the most different circRNA in buffalo and beef muscle *in vitro*. The results of *in vitro* experiments show that the overexpression of circEch1 inhibits the proliferation of bovine myoblasts but promotes differentiation; *in vivo* tests show that circEch1 stimulates skeletal muscle regeneration. In general, circEch1 induces myoblast differentiation and skeletal muscle regeneration (Huang et al., 2021). Although there are few studies in this area at present, we believe that with the development of research technology, the relationship between circRNAs and mRNA related to livestock muscle development will become more and more clear.

## Concluding Remarks and Perspectives

CircRNAs are a new regulator of muscle development. However, at present, the functional annotation of circRNAs is mainly to predict and analyze source coding genes or possible miRNA binding sites. In this review, we further discuss the relationship between circRNAs and muscle development of livestock by reviewing the discovery, classification, formation, characteristics, biological function, and degradation pathway of circRNAs. It is obvious that most studies focus on circRNAs guiding muscle development through the ceRNA co-expression regulation network. In addition, some studies confirm that circRNAs can regulate muscle development in livestock in transcription and translation, and only a few studies show that circRNAs can directly regulate muscle development in livestock. There is no doubt that our point of view plays a directional role in the study of muscle development in domestic animals; at the same time, we believe that there are other ways for circRNAs to regulate muscle development in livestock.

Up to now, the study of intracellular circRNAs made people further realize the complexity of eukaryotic gene expression regulation. In-depth study of the formation and types of circRNAs and the mechanism of action with target genes and exploring its biological function is of great significance for understanding the growth and development of organisms and disease treatment. Due to the variety of circRNAs, the diversity of action modes, and the constraints of research methods, people still need some time to clarify these genes and their regulatory mechanisms. However, as a kind of non-coding RNA discovered in the post-genome era, it effectively enriches the research model of gene expression regulation. We think that, with the rapid development of modern molecular biology technology, new generation sequencing technology, and bioinformation analysis technology, more and more new circRNAs will be discovered in the future, and people will study its function more and more deeply.

## Author Contributions

ZY and TH: conceptualization and writing-original draft and supervising, reviewing, and editing. ZY, TH, and QC: editing. All authors contributed to the article and approved the submitted version.

## Conflict of Interest

The authors declare that the research was conducted in the absence of any commercial or financial relationships that could be construed as a potential conflict of interest.

## Abbreviations

ceRNA: circRNAs-miRNAs-mRNAs
circRNAs: circular RNAs
FoxO1: Forkhead box transcription Factor O1
IGF-I: Insulin-like growth factor-I
IGF-II: Insulin-like growth factor-II
MEF2A-2D: myocyte enhancer factor2A-D
MIRs: Mammalian-Wide Interspersed Repeats
miRNAs: microRNAs
MRF-4: myogenic regulatory factor-4
MSTN: Myostatin
mTOR: Mammalian target of rapamycinMyf-5: myoblast regulatory factor family myogenic factor-5
Myhc: myosin heavy chain
MyoD: myogenic differentiation antigen
MyoG: myogenin
Pax: paired box
RBP: RNA binding protein
RNA-Seq: RNA sequencing.

## References

[B1] Abdelmohsen K. Panda A. C. De S. Grammatikakis I. Kim J. Ding J. (2015). Circular RNAs in monkey muscle: age-dependent changes. *Aging (Albany N.Y.)* 7 903–910. 10.18632/aging.100834 26546448 PMC4694061

[B2] Berger J. Hall T. E. Currie P. D. (2015). Novel transgenic lines to label sarcolemma and myofibrils of the musculature. *Zebrafish* 12 124–125. 10.1089/zeb.2014.1065 25554853

[B3] Burd C. E. Jeck W. R. Liu Y. Sanoff H. K. Wang Z. Sharpless N. E. (2010). Expression of linear and novel circular forms of an INK4/ARF-associated non-coding RNA correlates with atherosclerosis risk. *Plos Genet*. 6:e1001233–e1001247. 10.1371/journal.pgen.1001233 21151960 PMC2996334

[B4] Cao Y. You S. Yao Y. Liu Z. J. Hazi W. Li C. Y. (2018). Expression profiles of circular RNAs in sheep skeletal muscle. *Asian Austr. J. Anim*. 31 1550–1557. 10.5713/ajas.17.0563 PMC6127590 29642686

[B5] Chen B. You W. Wang Y. Shan T. (2020). The regulatory role of Myomaker and Myomixer-Myomerger-Minion in muscle development and regeneration. *Cell. Mol. Life Sci*. 77 1551–1569. 10.1007/s00018-019-03341-9 31642939 PMC11105057

[B6] Chen L. L. Yang L. (2015). Regulation of circRNA biogenesis. *RNA Biol*. 12 381–388. 10.1080/15476286.2015.1020271 25746834 PMC4615371

[B7] Chen P. R. Suh Y. Shin S. Woodfint R. M. Hwang S. Lee K. (2019). Exogenous expression of an alternative splicing variant of Myostatin prompts leg muscle fiber hyperplasia in Japanese quail. *Int. J. Mol. Sci*. 20 4617–4633. 10.3390/ijms20184617 31540432 PMC6770055

[B8] Chen S. Huang V. Xu X. Livingstone J. Soares F. Jeon J. (2019). Widespread and functional RNA circularization in localized prostate cancer. *Cell* 176 831–843. 10.1016/j.cell.2019.01.025 30735634

[B9] Christ B. Brand S. B. (2004). Limb muscle development. *Int. J. Dev. Biol*. 46 905–914. 10.1079/IVP2001258 12455628

[B10] Cocquerelle C. Mascrez B. Hétuin D. Bailleul B. (1993). Mis-splicing yields circular RNA molecules. *FASEB J*. 7 155–160. 10.1096/fasebj.7.1.7678559 7678559

[B11] Cook J. R. MacIntyre D. A. Samara E. Kim S. H. Singh N. Johnson M. R. (2015). Exogenous oxytocin modulates human myometrial microRNAs. *Am. J. Obstet. Gynecol*. 213 65–65. 10.1016/j.ajog.2015.03.015 25757635

[B12] Danan M. Schwartz S. Edelheit S. Sorek R. (2012). Transcriptome-wide discovery of circular RNAs in Archaea. *Nucleic Acids Res*. 40 3131–3142. 10.1093/nar/gkr1009 22140119 PMC3326292

[B13] Das A. Das A. Das D. Abdelmohsen K. Panda A. C. (2020). Circular RNAs in myogenesis. *BBA Gene Regul. Mech.* 1863 194372–194378. 10.1016/j.bbagrm.2019.02.011 30946990 PMC6773529

[B14] Das A. Rout P. K. Gorospe M. Panda A. C. (2019a). Past, present, and future of circRNAs. *EMBO J*. 38 e100836–e100848. 10.3390/ijms20163988 31343080 PMC6694216

[B15] Das A. Rout P. K. Gorospe M. Panda A. C. (2019b). Rolling circle cDNA synthesis uncovers circular RNA splice variants. *Int. J. Mol. Sci.* 20 3988–3999. 10.3390/ijms20163988 PMC6721031 31426285

[B16] Doynova M. D. Markworth J. F. Cameron-Smith D. Vickers M. H. O’Sullivan J. M. (2017). Linkages between changes in the 3D organization of the genome and transcription during myotube differentiation in vitro. *Skelet. Muscle* 7 5–14. 10.1186/s13395-017-0122-1 28381300 PMC5382473

[B17] Du W. W. Yang W. Li X. Awan F. M. Yang Z. Fang L. (2018). A circular RNA circ-DNMT1 enhances breast cancer progression by activating autophagy. *Oncogene* 37 5829–5842. 10.1038/s41388-018-0369-y 29973691

[B18] Ebbesen K. K. Hansen T. B. Kjems J. (2017). Insights into circular RNA biology. *RNA Biol*. 14 1035–1045. 10.1080/15476286.2016.1271524 27982727 PMC5680708

[B19] Fischer J. W. Busa V. F. Shao Y. Leung A. (2020). Structure-mediated RNA Decay by UPF1 and G3BP1. *Mol. Cell* 78 70–84. 10.1016/j.molcel.2020.01.021 32017897 PMC8055448

[B20] Geng H. H. Li R. Su Y. M. Xiao J. Pan M. Cai X. X. (2016). The circular RNA Cdr1as promotes myocardial infarction by mediating the regulation of miR-7a on its target genes expression. *PLoS One* 11:e0151753–e0151769. 10.1371/journal.pone.0151753 26998750 PMC4801407

[B21] Granados-Riveron J. T. Aquino-Jarquin G. (2016). The complexity of the translation ability of circRNAs. *BBA Gene Regul. Mech*. 1859 1245–1251. 10.1016/j.bbagrm.2016.07.009 27449861

[B22] Greco S. De Simone M. Colussi C. Zaccagnini G. Fasanaro P. Pescatori M. (2009). Common micro-RNA signature in skeletal muscle damage and regeneration induced by Duchenne muscular dystrophy and acute ischemia. *FASEB J*. 23 3335–3346. 10.1096/fj.08-128579 19528256

[B23] Hansen T. B. Wiklund E. D. Bramsen J. B. Villadsen S. B. Statham A. L. Clark S. J. (2011). miRNA-dependent gene silencing involving Ago2-mediated cleavage of a circular antisense RNA. *EMBO J*. 30 4414–4422. 10.1038/emboj.2011.359 21964070 PMC3230379

[B24] Hernández-Hernández J. M. García-González E. G. Brun C. E. Rudnicki M. A. (2017). The myogenic regulatory factors, determinants of muscle development, cell identity and regeneration. *Semin. Cell Dev. Biol*. 72 10–18. 10.1016/j.semcdb.2017.11.010 29127045 PMC5723221

[B25] Holdt L. M. Stahringer A. Sass K. Pichler G. Kulak N. A. Wilfert W. (2016). Circular non-coding RNA ANRIL modulates ribosomal RNA maturation and atherosclerosis in humans. *Nat. Commun*. 7:12429. 10.1038/ncomms12429 27539542 PMC4992165

[B26] Hsu M. T. Coca-Prados M. (1979). Electron microscopic evidence for the circular form of RNA in the cytoplasm of eukaryotic cells. *Nature* 280 339–340. 10.1038/280339a0 460409

[B27] Huang K. W. Chen M. J. Zhong D. D. Luo X. Feng T. Song M. M. (2021). Circular RNA profifiling reveals an abundant circEch1 that promotes myogenesis and differentiation of bovine skeletal muscle. *J. Agric. Food Chem.* 69 592–601. 10.1021/acs.jafc.0c06400 33346638

[B28] Humphreys D. T. Fossat N. Demuth M. Tam P. Ho J. (2019). Ularcirc: visualization and enhanced analysis of circular RNAs via back and canonical forward splicing. *Nucleic Acids Res*. 47 e123–e139. 10.1093/nar/gkz718 31435647 PMC6846653

[B29] Irie T. Shum R. Deni I. Hunkele A. Le Rouzic V. Xu J. (2019). Identification of abundant and evolutionarily conserved opioid receptor circular RNAs in the nervous system modulated by morphine. *Mol. Pharmacol*. 96 247–258. 10.1124/mol.118.113977 31243060 PMC6666384

[B30] Janssen I. Heymsfield S. B. Wang Z. M. Ross R. (2000). Skeletal muscle mass and distribution in 468 men and women aged 18–88 yr. *J. Appl. Physiol*. 89 81–88. 10.1152/jappl 10904038

[B31] Kos A. Dijkema R. Arnberg A. C. van der Meide P. H. Schellekens H. (1986). The hepatitis delta (δ) virus possesses a circular RNA. *Nature* 323 558–560. 10.1038/323558a0 2429192

[B32] Kotake Y. Nakagawa T. Kitagawa K. Suzuki S. Liu N. Kitagawa M. (2011). Long non-coding RNA ANRIL is required for the PRC2 recruitment to and silencing of p15 INK4B tumor suppressor gene. *Oncogene* 30 1956–1962. 10.1038/onc.2010.568 21151178 PMC3230933

[B33] Kyei B. Li L. Yang L. Zhan S. Zhang H. (2020). CDR1as/miRNAs-related regulatory mechanisms in muscle development and diseases. *Gene* 730 144315–144322. 10.1016/j.gene.2019.144315 31904497

[B34] Lasda E. Parker R. (2014). Circular RNAs: diversity of form and function. *RNA* 20 1829–1842. 10.1261/rna.047126.114 25404635 PMC4238349

[B35] Legnini I. Di Timoteo G. Rossi F. Morlando M. Briganti F. Sthandier O. (2017). Circ-ZNF609 is a circular RNA that can be translated and functions in myogenesis. *Mol. Cell* 66 22–37. 10.1016/j.molcel.2017.02.017 28344082 PMC5387670

[B36] Li B. Yin D. Li P. Zhang Z. Zhang X. Li H. (2020). Profiling and functional analysis of circular RNAs in porcine fast and slow muscles. *Front. Cell Dev. Biol*. 8:322–324. 10.3389/fcell.2020.00322 32528948 PMC7264268

[B37] Li C. Li X. Yao Y. Ma Q. Ni W. Zhang X. (2017). Genome-wide analysis of circular RNAs in prenatal and postnatal muscle of sheep. *Oncotarget* 8 97165–97177. 10.18632/oncotarget.21835 29228601 PMC5722553

[B38] Li D. Li Z. Yang Y. Zeng X. Li Y. Du X. (2020). Circular RNAs as biomarkers and therapeutic targets in environmental chemical exposure-related diseases. *Environ. Res*. 180 108825–108833. 10.1016/j.envres.2019.108825 31683121

[B39] Li H. Wei X. Yang J. Dong D. Hao D. Huang Y. (2018a). circFGFR4 promotes differentiation of myoblasts via binding miR-107 to relieve its inhibition of Wnt3a. *Mol. Ther. Nucleic Acids* 11 272–283. 10.1016/j.omtn.2018.02.012 29858062 PMC5992882

[B40] Li H. Yang J. Wei X. Song C. Dong D. Huang Y. (2018b). CircFUT10 reduces proliferation and facilitates differentiation of myoblasts by sponging miR-133a. *J. Cell Physiol*. 233 4643–4651. 10.1002/jcp.26230 29044517

[B41] Li L. Chen Y. Nie L. Ding X. Zhang X. Zhao W. (2019). MyoD-induced circular RNA CDR1as promotes myogenic differentiation of skeletal muscle satellite cells. *BBA Gene Regul. Mech*. 1862 807–821. 10.1016/j.bbagrm.2019.07.001 31323434

[B42] Li X. F. Lytton J. (1999). A circularized sodium-calcium exchanger exon 2 transcript. *J. Biol. Chem*. 274 8153–8160. 10075718 10.1074/jbc.274.12.8153

[B43] Li X. Liu S. Zhang L. Issaian A. Hill R. C. Espinosa S. (2019). A unified mechanism for intron and exon definition and back-splicing. *Nature* 573 375–380. 10.1038/s41586-019-1523-6 31485080 PMC6939996

[B44] Li X. Xie S. Qian L. Cai C. Bi H. Cui W. (2020). Identification of genes related to skeletal muscle growth and development by integrated analysis of transcriptome and proteome in myostatin-edited Meishan pigs. *J. Proteomics* 213 103628–103639. 10.1016/j.jprot.2019.103628 31881351

[B45] Liang D. Tatomer D. C. Luo Z. Wu H. Yang L. Chen L. L. (2017). The output of protein-coding genes shifts to circular RNAs when the pre-mRNA processing machinery is limiting. *Mol. Cell* 68 940–954. 10.1016/j.molcel.2017.10.034 29174924 PMC5728686

[B46] Liang G. Yang Y. Niu G. Tang Z. Li K. (2017a). Circular RNA profiling reveals an abundant circLMO7 that regulates myoblasts differentiation and survival by sponging miR-378a-3p. *Cell Death Dis*. 8 e3153–e3165. 10.1093/dnares/dsx022 29072698 PMC5680912

[B47] Liang G. Yang Y. Niu G. Tang Z. Li K. (2017b). Genome-wide profiling of *Sus scrofa* circular RNAs across nine organs and three developmental stages. *DNA Res*. 24 523–535. 28575165 10.1093/dnares/dsx022 PMC5737845

[B48] Liang W. C. Wong C. W. Liang P. P. Shi M. Cao Y. Rao S. T. (2019). Translation of the circular RNA circβ-catenin promotes liver cancer cell growth through activation of the Wnt pathway. *Genome Biol*. 20:84. 10.1186/s13059-019-1685-4 31027518 PMC6486691

[B49] Ling Y. Zheng Q. Zhu L. Xu L. Sui M. Zhang Y. (2020). Trend analysis of the role of circular RNA in goat skeletal muscle development. *BMC Genomics* 21:220. 10.1186/s12864-020-6649-2 32151242 PMC7063781

[B50] Liu B. Song F. Yang Q. Zhou Y. Shao C. Shen Y. (2019). Characterization of tissue-specific biomarkers with the expression of circRNAs in forensically relevant body fluids. *Int. J. Legal. Med*. 133 1321–1331. 10.1007/s00414-019-02027-y 30810820

[B51] Liu C. X. Li X. Nan F. Jiang S. Gao X. Guo S. K. (2019). Structure and degradation of circular RNAs regulate PKR activation in innate immunity. *Cell* 177 865–880. 10.1016/j.cell.2019.03.046 31031002

[B52] Liu X. Hu Z. Zhou J. Tian C. Tian G. He M. (2020a). Interior circular RNA. *RNA Biol.* 17 87–97. 10.1080/15476286.2019.1669391 31532701 PMC6948956

[B53] Liu X. Wang X. Li J. Hu S. Deng Y. Yin H. (2020b). The identification of mecciRNAs and their roles in mitochondrial entry of proteins. *Sci. China Life Sci*. 63 1429–1449. 10.1007/s11427-020-1631-9 32048164

[B54] McPherron A. C. Lee S. J. (2002). Suppression of body fat accumulation in myostatin-deficient mice. *J. Clin. Invest*. 109 595–601. 10.1172/JCI13562 11877467 PMC150888

[B55] Memczak S. Jens M. Elefsinioti A. Torti F. Krueger J. Rybak A. (2013). Circular RNAs are a large class of animal RNAs with regulatory potency. *Nature* 495 333–338. 10.1038/nature11928 23446348

[B56] Molkentin J. D. Olson E. N. (1996). Defining the regulatory networks for muscle development. *Curr. Opin. Genet. Dev*. 6 445–453. 10.1016/s0959-437x(96)80066-9 8791524

[B57] Naqvi A. S. Asnani M. Black K. L. Hayer K. E. Taylor D. Thomas-Tikhonenko A. (2019). The role of SRSF3 splicing factor in generating circular RNAs. *bioRxiv* [Preprint] 799700. 10.1101/799700

[B58] Ng P. C. Chan K. Y. Leung K. T. Tam Y. H. Ma T. P. Lam H. S. (2015). Comparative MiRNA expressional profiles and molecular networks in human small bowel tissues of necrotizing enterocolitis and spontaneous intestinal perforation. *PLoS One* 10:e0135737–e0135753. 10.1371/journal.pone.0135737 26274503 PMC4537110

[B59] Ouyang H. Chen X. Wang Z. Yu J. Jia X. Li Z. (2018). Circular RNAs are abundant and dynamically expressed during embryonic muscle development in chickens. *DNA Res*. 25 71–86. 10.1093/dnares/dsx039 29036326 PMC5824844

[B60] Pagliarini V. Jolly A. Bielli P. Di Rosa V. De la Grange P. Sette C. (2020). Sam68 binds Alu-rich introns in SMN and promotes pre-mRNA circularization. *Nucleic Acids Res*. 48 633–645. 10.1093/nar/gkz1117 31777926 PMC6954450

[B61] Pamudurti N. R. Bartok O. Jens M. Ashwal-Fluss R. Stottmeister C. Ruhe L. (2017). Translation of circRNAs. *Mol. Cell* 66 9–21. 10.1016/j.molcel.2017.02.021 28344080 PMC5387669

[B62] Pandey P. R. Yang J. H. Tsitsipatis D. Panda A. C. Gorospe M. (2020). circSamd4 represses myogenic transcriptional activity of PUR proteins. *Nucleic Acids Res.* 48 3789–3805. 10.1093/nar/gkaa035 31980816 PMC7144931

[B63] Park O. H. Ha H. Lee Y. Boo S. H. Kwon D. H. Song H. K. (2019). Endoribonucleolytic cleavage of m6A-containing RNAs by RNase P/MRP complex. *Mol. Cell* 74 494–507. 10.1016/j.molcel.2019.02.034 30930054

[B64] Patop I. L. Wust S. Kadener S. (2019). Past, present, and future of circRNAs. *Embo. J.* 38:e100836. 10.15252/embj.2018100836 31343080 PMC6694216

[B65] Peng S. Song C. Li H. Cao X. Ma Y. Wang X. (2019). Circular RNA SNX29 sponges miR-744 to regulate proliferation and differentiation of myoblasts by activating the Wnt5a/Ca2+ signaling pathway. *Mol. Ther. Nucleic Acids* 16 481–493. 10.1016/j.omtn.2019.03.009 31051333 PMC6495097

[B66] Qian D. Y. Yan G. B. Bai B. Chen Y. Zhang S. J. Yao Y. C. (2017). Differential circRNA expression profiles during the BMP2-induced osteogenic differentiation of MC3T3-E1 cells. *Biomed. Pharmacother*. 90 492–499. 10.1016/j.biopha.2017.03.051 28395271

[B67] Qu S. Yang X. Li X. Wang J. Gao Y. Shang R. (2015). Circular RNA: a new star of noncoding RNAs. *Cancer Lett*. 365 141–148. 10.1016/j.canlet.2015.06.003 26052092

[B68] Rong D. Sun H. Li Z. Liu S. Dong C. Fu K. (2017). An emerging function of circRNA-miRNAs-mRNA axis in human diseases. *Oncotarget* 8 73271–73281. 10.18632/oncotarget.19154 29069868 PMC5641211

[B69] Salzman J. Gawad C. Wang P. L. Lacayo N. Brown P. O. (2012). Circular RNAs are the predominant transcript isoform from hundreds of human genes in diverse cell types. *PLos One* 7:e30733–e30744. 10.1371/journal.pone.0030733 22319583 PMC3270023

[B70] Sang M. Meng L. Sang Y. Liu S. Ding P. Ju Y. (2018). Circular RNA ciRS-7 accelerates ESCC progression through acting as a miR-876-5p sponge to enhance MAGE-A family expression. *Cancer Lett*. 426 37–46. 10.1016/j.canlet.2018.03.049 29635069

[B71] Sanger H. L. Klotz G. Riesner D. Gross H. J. Kleinschmidt A. K. (1976). Viroids are single-stranded covalently closed circular RNA molecules existing as highly base-paired rod-like structures. *Proc. Natl. Acad. Sci. U.S.A.* 73 3852–3856. 10.1073/pnas.73.11.3852 1069269 PMC431239

[B72] Shen L. Gan M. Tang Q. Tang G. Jiang Y. Li M. (2019). Comprehensive analysis of lncRNAs and circRNAs reveals the metabolic specialization in oxidative and glycolytic skeletal muscles. *Int. J. Mol. Sci*. 20 2855–2872. 10.3390/ijms20122855 31212733 PMC6627206

[B73] Silva W. J. Graça F. A. Cruz A. Silvestre J. G. Labeit S. Miyabara E. H. (2019). miR-29c improves skeletal muscle mass and function throughout myocyte proliferation and differentiation and by repressing atrophy-related genes. *Acta Physlol*. 226 e13278–e13296. 10.1111/apha.13278 30943315 PMC6900115

[B74] Song Z. B. Liu Y. M. Fang X. B. Xie M. S. Ma Z. Y. Zhong Z. G. (2020). Comprehensive analysis of the expression profile of circRNAs and their predicted protein-coding ability in the muscle of mdx mice. *Funct. Integer. Genomic* 20 397–407. 10.1007/s10142-019-00724-w 31736012

[B75] Sun J. Xie M. Huang Z. Li H. Chen T. Sun R. (2017). Integrated analysis of non-coding RNA and mRNA expression profiles of 2 pig breeds differing in muscle traits. *J. Anim. Sci*. 95 1092–1103. 10.2527/jas.2016.0867 28380516

[B76] Suzuki H. Tsukahara T. (2014). A view of pre-mRNA splicing from RNase R resistant RNAs. *Int. J. Mol. Sci*. 15 9331–9342. 10.3390/ijms15069331 24865493 PMC4100097

[B77] Suzuki H. Zuo Y. Wang J. Zhang M. Q. Malhotra A. Mayeda A. (2006). Characterization of RNase R-digested cellular RNA source that consists of lariat and circular RNAs from pre-mRNA splicing. *Nucleic Acids Res*. 34 e63–e69. 10.1093/nar/gkl151 16682442 PMC1458517

[B78] Tan S. Gou Q. Pu W. Guo C. Yang Y. Wu K. (2018). Circular RNA F-circEA produced from EML4-ALK fusion gene as a novel liquid biopsy biomarker for non-small cell lung cancer. *Cell Res*. 28 693–695. 10.1038/s41422-018-0033-7 29628502 PMC5993747

[B79] Tang C. Xie Y. Yu T. Liu N. Wang Z. Woolsey R. J. (2020). m6A-dependent biogenesis of circular RNAs in male germ cells. *Cell Res*. 30 211–228. 10.1038/s41422-020-0279-8 32047269 PMC7054367

[B80] Tuck A. C. Rankova A. Arpat A. B. Liechti L. A. Hess D. Iesmantavicius V. (2020). Mammalian RNA decay pathways are highly specialized and widely linked to translation. *Mol. Cell* 77 1222–1236. 10.1016/j.molcel.2020.01.007 32048998 PMC7083229

[B81] von Maltzahn J. Chang N. C. Bentzinger C. F. Rudnicki M. A. (2012). Wnt signaling in myogenesis. *Trends Cell Biol*. 22 602–609. 10.1016/j.tcb.2012.07.008 22944199 PMC3479319

[B82] Wan H. Zhu J. Su G. Liu Y. Hua L. Hu L. (2016). Dietary supplementation with β-hydroxy-β-methylbutyrate calcium during the early postnatal period accelerates skeletal muscle fibre growth and maturity in intra-uterine growth-retarded and normal-birth-weight piglets. *Br. J. Nutr*. 115 1360–1369. 10.1017/S0007114516000465 26917333

[B83] Wang F. Nazarali A. J. Ji S. (2016). Circular RNAs as potential biomarkers for cancer diagnosis and therapy. *Am. J. Cancer Res.* 6 1167–1176. 27429839 PMC4937728

[B84] Wang X. Cao X. Dong D. Shen X. Cheng J. Jiang R. (2019). Circular RNA TTN acts as a miR-432 sponge to facilitate proliferation and differentiation of myoblasts via the IGF2/PI3K/AKT signaling pathway. *Mol. Ther. Nucleic Acids* 18 966–980. 10.1016/j.omtn.2019.10.019 31770673 PMC6881651

[B85] Wang Y. Wang Z. (2015). Efficient backsplicing produces translatable circular mRNAs. *RNA Publ RNA Soc*. 21 172–179. 10.1261/rna.048272.114 25449546 PMC4338345

[B86] Westholm J. O. Miura P. Olson S. Shenker S. Joseph B. Sanfilippo P. (2014). Genome-wide analysis of drosophila circular RNAs reveals their structural and sequence properties and age-dependent neural accumulation. *Cell Rep*. 9 966–1980. 10.1016/j.celrep.2014.10.062 25544350 PMC4279448

[B87] Wei X. Li H. Yang J. Hao D. Dong D. Huang Y. (2017). Circular RNA profiling reveals an abundant circLMO7 that regulates myoblasts differentiation and survival by sponging miR-378a-3p. *Cell Death Dis*. 8 e3153–e3165. 10.1038/cddis.2017.541 29072698 PMC5680912

[B88] Wei X. Li H. Zhang B. Li C. Dong D. Lan X. (2016). miR-378a-3p promotes differentiation and inhibits proliferation of myoblasts by targeting HDAC4 in skeletal muscle development. *RNA Biol*. 13 1300–1309. 10.1080/15476286.2016.1239008 27661135 PMC5207390

[B89] Wu K. Liao X. Gong Y. He J. Zhou J. K. Tan S. (2019). Circular RNA F-circSR derived from SLC34A2-ROS1 fusion gene promotes cell migration in non-small cell lung cancer. *Mol. Cancer*. 18 98–103. 10.1186/s12943-019-1028-9 31118036 PMC6530145

[B90] Yan X. M. Zhang Z. Meng Y. Li H. B. Gao L. Luo D. (2020). Genome-wide identification and analysis of circular RNAs differentially expressed in the longissimus dorsi between Kazakh cattle and Xinjiang brown cattle. *PEER J*. 8 8646–8662. 10.7717/peerj.8646 PMC7081781 32211228

[B91] Yang Y. B. Gao X. Y. Zhang M. L. Yan S. Sun C. J. Xiao F. Z. (2018). Novel role of FBXW7 circular RNA in repressing Glioma Tumorigenesis. *J. Natl. Cancer Inst.* 110 304–315. 10.1093/jnci/djx166 28903484 PMC6019044

[B92] Yang Z. G. Awan F. M. Du W. W. Zeng Y. Lyu J. Wu D. (2017). The circular RNA interacts with STAT3, increasing its nuclear translocation and wound repair by modulating Dnmt3a and miR-17 function. *Mol. Ther*. 25 2062–2074. 10.1016/j.ymthe.2017.05.022 28676341 PMC5589065

[B93] Yin H. D. Shen X. X. Zhao J. Cao X. A. He H. R. Han S. S. (2020). Circular RNA CircFAM188B encodes a protein that regulates proliferation and differentiation of chicken skeletal muscle satellite cells. *Front. Cell Dev. Biol.* 8:522588. 10.3389/fcell.2020.522588 33240871 PMC7677141

[B94] Yoshimoto R. Rahimi K. Hansen T. B. Kjems J. Mayeda A. (2020). Biosynthesis of circular RNA ciRS-7/CDR1as is mediated by mammalian-wide interspersed repeats (MIRs). *Iscience* 23 101345–101359. 10.1016/j.isci.2020.101345 32683316 PMC7371899

[B95] Yue B. Wang J. Ru W. Wu J. Cao X. Yang H. (2020). The circular RNA circHUWE1 sponges the miR-29b-AKT3 axis to regulate myoblast development. *Mol. Ther. Nucleic Acids* 19 1086–1097. 10.1016/j.omtn.2019.12.039 32045877 PMC7015828

[B96] Zeng K. Chen X. Xu M. Liu X. Hu X. Xu T. (2018). CircHIPK3 promotes colorectal cancer growth and metastasis by sponging miR-7. *Cell Death Dis*. 9:417. 10.1038/s41419-018-0454-8 29549306 PMC5856798

[B97] Zhang P. Xu H. Li R. Wu W. Chao Z. Li C. (2018). Assessment of myoblast circular RNA dynamics and its correlation with miRNA during myogenic differentiation. *Biochem. Cell Biol*. 99 211–218. 10.1016/j.biocel.2018.04.016 29684477

[B98] Zhang P. P. Chao Z. Zhang R. Ding R. Q. Wang Y. L. Wu W. (2019). Circular RNA Regulation of Myogenesis. *Cells* 8 885–897. 10.3390/cells8080885 31412632 PMC6721685

[B99] Zhang X. O. Wang H. B. Zhang Y. Lu X. Chen L. L. Yang L. (2014). Complementary sequence-mediated exon circularization. *Cell* 159 134–147. 10.1016/j.cell.2014.09.001 25242744

[B100] Zhang Y. Zhang X. O. Chen T. Xiang J. F. Yin Q. F. Xing Y. H. (2013). Circular intronic long noncoding RNAs. *Mol. Cell* 51 792–806. 10.1016/j.molcel.2013.08.017 24035497

[B101] Zhao J. Wu J. Xu T. Yang Q. He J. Song X. (2018). IRESfinder: identifying RNA internal ribosome entry site in eukaryotic cell using framed k-mer features. *J. Genet. Genomics* 45 403–406. 10.1016/j.jgg.2018.07.006 30054216

[B102] Zhao W. Cui Y. Liu L. Qi X. Liu J. Ma S. (2020). Splicing factor derived circular RNA circUHRF1 accelerates oral squamous cell carcinoma tumorigenesis via feedback loop. *Cell Death Differ*. 27 919–933. 10.1038/s41418-019-0423-5 31570856 PMC7206121

